# Deep learning models for preoperative T-stage assessment in rectal cancer using MRI: exploring the impact of rectal filling

**DOI:** 10.3389/fmed.2023.1326324

**Published:** 2023-11-29

**Authors:** Chang Tian, Xiaolu Ma, Haidi Lu, Qian Wang, Chengwei Shao, Yuan Yuan, Fu Shen

**Affiliations:** ^1^School of Information Science and Technology and School of Biomedical Engineering, ShanghaiTech University, Shanghai, China; ^2^Department of Radiology, Changhai Hospital, The Navy Medical University, Shanghai, China; ^3^School of Biomedical Engineering, ShanghaiTech University, Shanghai, China

**Keywords:** rectal cancer, T staging, MRI, deep learning, rectal filling

## Abstract

**Background:**

The objective of this study was twofold: firstly, to develop a convolutional neural network (CNN) for automatic segmentation of rectal cancer (RC) lesions, and secondly, to construct classification models to differentiate between different T-stages of RC. Additionally, it was attempted to investigate the potential benefits of rectal filling in improving the performance of deep learning (DL) models.

**Methods:**

A retrospective study was conducted, including 317 consecutive patients with RC who underwent MRI scans. The datasets were randomly divided into a training set (*n* = 265) and a test set (*n* = 52). Initially, an automatic segmentation model based on T2-weighted imaging (T2WI) was constructed using nn-UNet. The performance of the model was evaluated using the dice similarity coefficient (DSC), the 95th percentile Hausdorff distance (HD95), and the average surface distance (ASD). Subsequently, three types of DL-models were constructed: Model 1 trained on the total training dataset, Model 2 trained on the rectal-filling dataset, and Model 3 trained on the non-filling dataset. The diagnostic values were evaluated and compared using receiver operating characteristic (ROC) curve analysis, confusion matrix, net reclassification index (NRI), and decision curve analysis (DCA).

**Results:**

The automatic segmentation showed excellent performance. The rectal-filling dataset exhibited superior results in terms of DSC and ASD (*p* = 0.006 and 0.017). The DL-models demonstrated significantly superior classification performance to the subjective evaluation in predicting T-stages for all test datasets (all *p* < 0.05). Among the models, Model 1 showcased the highest overall performance, with an area under the curve (AUC) of 0.958 and an accuracy of 0.962 in the filling test dataset.

**Conclusion:**

This study highlighted the utility of DL-based automatic segmentation and classification models for preoperative T-stage assessment of RC on T2WI, particularly in the rectal-filling dataset. Compared with subjective evaluation, the models exhibited superior performance, suggesting their noticeable potential for enhancing clinical diagnosis and treatment practices.

## Background

Colorectal cancer (CRC) stands as the second most prevalent contributor to cancer-related mortality in the United States. Projections for the year 2023 indicate that approximately 153,020 individuals will receive a diagnosis of CRC, and regrettably, 52,550 individuals will succumb to the disease. This includes a concerning subset of 19,550 cases and 3,750 deaths among individuals below the age of 50 years old (1). Rectal cancer (RC) is a subset of CRC, a disease that poses a grave risk to people’s lives. Rectal magnetic resonance imaging (MRI) has witnessed widespread utilization in the comprehensive assessment of RC, assuming a vital role in treatment planning for patients by facilitating accurate preoperative tumor staging. Within clinical practice, high-resolution T2-weighted imaging (HR-T2WI) has gained unanimous acceptance as the optimal approach for preoperative staging of RC (2). The precise preoperative differentiation between T1-2 and T3-4 stages in RC holds immense significance for clinicians in guiding individualized treatment strategies. The ability to discern which patients should undergo total mesorectal excision (TME) or receive neoadjuvant treatment while minimizing the risks of both over-treatment and under-treatment has become paramount (3, 4). However, the traditional approach to MRI staging relies heavily on the expertise and subjective evaluation of radiologists, leading to diminished repeatability and accuracy rates. This reliance poses significant challenges in achieving an accurate preoperative T-stage diagnosis for RC (5). Furthermore, a contentious issue surrounds the use of rectal distension during rectal MRI, specifically regarding whether the rectal lumen should be filled with fluid or gel (2–4). While the primary objective of rectal filling is to optimize lesion visualization and improve T-stage assessment on MRI, the question of its routine application remains unresolved due to a lack of robust evidence demonstrating substantial improvements in lesion conspicuity (3–5).

In recent years, radiomics has emerged as a potential method for addressing diverse clinical challenges, surpassing traditional methods in several studies. By leveraging high-throughput analysis to extract a multitude of quantitative features from medical images, radiomics approaches have demonstrated promising potential in the field of digestive tumors (6–17). However, the predominant methodologies in this domain typically involve manually determining the volume of the entire primary tumor. This process is not only arduous and time-consuming but also heavily reliant on the operator’s expertise, demanding a high level of proficiency (9, 16, 17).

Previous study yielded an initial finding indicating the development of two distinct radiomics models utilizing rectal HR-T2WI, both with and without rectal filling. These models were devised to evaluate the T staging of RC. Notably, our results demonstrated the superior performance of the radiomics model incorporating rectal filling in effectively distinguishing between T1-2 and T3 stages. This promising outcome suggests that the utilization of this model could offer valuable support in clinical decision-making when evaluating T-stage in RC patients (6).

Meanwhile, the deep learning (DL)-based method, as a novel technology, could significantly improve lesion automatic localization and segmentation, tumor diagnosis, staging, and prognosis prediction to facilitate treatment strategy, and could even greatly help radiologists work more efficiently and reduce their burden (18–20). Despite the considerable significance of T staging in RC, there exists a notable research gap regarding the validation and comparative analysis of MRI-based DL approaches specifically tailored for T staging evaluation, taking into account the presence or absence of rectal filling.

Therefore, in this study, we initially attempted to construct a convolutional neural network for the automatic localization and segmentation of RC lesions. Subsequently, we developed DL networks for the assessment of RC T-staging. Of utmost importance was our exploration of whether rectal filling could prove beneficial in guiding clinical decision-making for RC T stage evaluation.

## Methods

### Participants

This study followed the Declaration of Helsinki and had approval from the Ethics Committees of Changhai Hospital. Written informed consent was waived from all patients.

**Figure 1:**
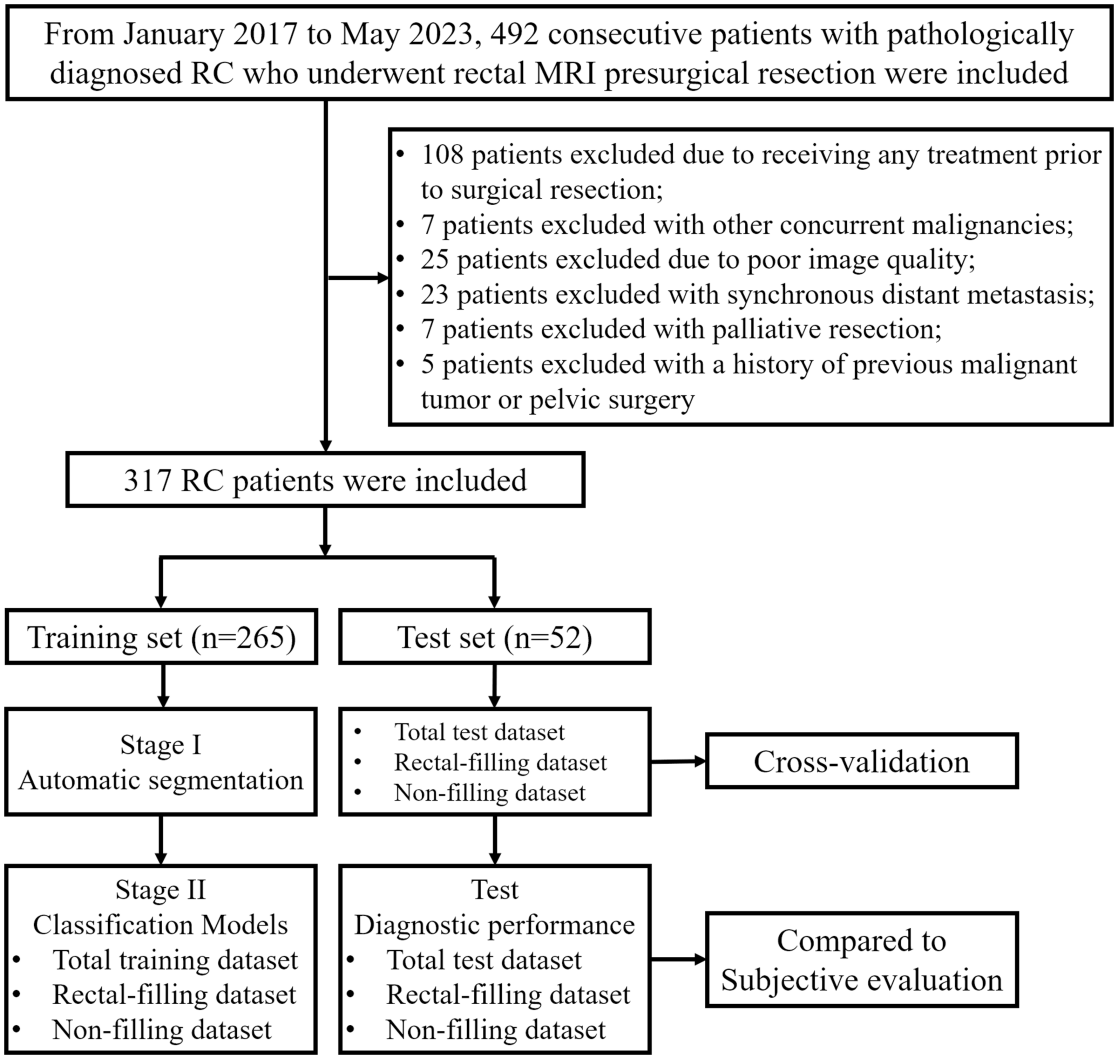
Flowchart of the study.

This retrospective trial enrolled a total of 492 consecutive patients with RC who underwent radical resection at Changhai Hospital between January 2017 and May 2023. The study’s inclusion criteria comprised the following: (1) confirmation of rectal adenocarcinoma through postoperative pathological examination; (2) presence of a single lesion; (3) baseline rectal magnetic resonance (MR) examination conducted within 14 days prior to surgical resection. Exclusion criteria included: (1) receipt of any local or systemic treatment prior to surgical resection, such as neoadjuvant chemoradiotherapy (*n* = 108); (2) concurrent diagnosis of other malignancies (*n* = 7); (3) poor image quality (*n* = 25); (4) synchronous distant metastasis (*n* = 23); (5) palliative resection (*n* = 7); (6) history of previous pelvic surgery (*n* = 5). Consequently, a total of 317 cases were included in the final analysis, as depicted in Figure 1.

### Clinicopathologic data

Patients’ demographic and clinicopathological data were retrospectively extracted from the clinicopathological databases. These data encompassed various factors, including sex, age, body mass index (BMI), histological differentiation, pathological T-stage, pathological N-stage, carcinoembryonic antigen (CEA) levels (with <5 ng/mL considered as negative), and carbohydrate antigen 19-9 (CA19-9) levels (with <37 U/mL considered as negative). These parameters were recorded concurrently with the baseline MRI examinations. Employing the criteria set forth by the National Comprehensive Cancer Network (NCCN) and American Joint Committee on Cancer (AJCC) staging system (21), the patients involved in the study were meticulously stratified into distinct cohorts, each characterized by their respective pathological T stages. Specifically, the T1-2 group encompassed individuals with tumors confined solely to the submucosal and muscularis propria layers. In contrast, the T3-4 group comprised patients with tumors that exhibited invasive growth beyond the confines of the muscularis propria.

### Image acquisition and analysis

Prior to the study, baseline rectal MRI scans were performed using 3.0 T MR systems, including the Siemens 3.0 T MAGNETOM Skyra MRI System, GE 3.0 T Discovery MR 750w, and Signa HDX System, coupled with a specialized phased array coil for enhanced imaging sensitivity. To ensure optimal image quality, intestinal cleaning was meticulously carried out through the administration of a 20 mL glycerin enema. Considering the possibility of contraindications, the administration of raceanisodamine hydrochloride, a commonly used agent, was deliberately omitted. As part of the routine imaging procedure, oblique axial HR-T2WI was conducted with careful consideration of the orientation perpendicular to the long axis of the rectum, encompassing the region of interest (ROI). Notably, detailed information regarding the parameters employed for HR-T2WI, which played a pivotal role in the subsequent analysis, can be found in Supplementary Table S1.

Within the filling group, patients underwent a baseline MRI with rectal filling, involving the administration of warm ultrasound (US) transmission gel to achieve rectal distention. Prior to acquiring the oblique axial HR-T2W images, the volume of gel used for rectal filling was tailored based on the endoscopic evaluation of tumor location. Specifically, 60–80 mL of gel was administered for lesions situated in the lower and middle rectum, while 80–100 mL was utilized for lesions in the upper rectum (22). Conversely, in the non-filling group, rectal distention using US gel was omitted during the baseline MRI procedure.

Subjective evaluation and ROI delineation were performed by 3 radiologists with systematic training, including FS, HL, and YY with 15, 11, and 13 years of experience in MR diagnosis, respectively, who were blinded to pathological data. A subjective classification task was assigned to the experts, requiring them to categorize each lesion as either T1-2 or T3-4 based on the established TNM staging system. Interobserver agreement for MR T-staging among the three radiologists was calculated. To facilitate accurate lesion segmentation, the ROI encompassing the entire rectal lesion was manually delineated in a meticulous slice-by-slice manner on the T2WI using ITK-SNAP 4.0.0 software^1^. The delineated borders, representing the ground truth (GT), were meticulously determined by consensus among the experts. In cases of any discrepancies or differences of opinion, a thorough discussion ensued until a consensus was reached, requiring the agreement of at least two experts.

### Dataset and pre-processing

A dataset comprising 317 MRI scans of RC and their corresponding T-stage labels was extracted and subsequently divided into a training set (*n* = 265) and a test set (*n* = 52) using a random allocation in a ratio of approximately 5:1. For the segmentation task, we utilized the preprocessing pipeline of nn-UNet (23–25), which could select the suitable data fingerprint automatically. We adopted the data preprocessing strategy through data fingerprint information, including resampling strategy, cropping area size, gray value distribution, etc. information, thus forming a so-called “configuration plan.” While for the classification task, to ensure consistency, all images underwent preprocessing too from the “configuration plan,” including resampling to a target spacing of [0.36, 0.36, 0.36] mm. Additionally, the size of each imaging scan was adjusted by cropping or padding to achieve a uniform dimension of 384 × 384 × 64.

### DL model construction

The U-Net architecture (23), introduced in 2015 as an Encoder–Decoder model (24), made a significant impact in the field of medical segmentation, generating widespread enthusiasm. Subsequent studies have primarily concentrated on maximizing the potential of U-Net and enhancing its performance through various modifications. Presently, UNet-like Encoder–Decoder architectures remain robust and highly regarded in the field. One notable variant, nn-UNet (25), exemplifies the remarkable qualities of U-Net as a self-configuring approach and pipeline for DL-based biomedical image segmentation, consistently delivering exceptional results.

**Figure 2:**
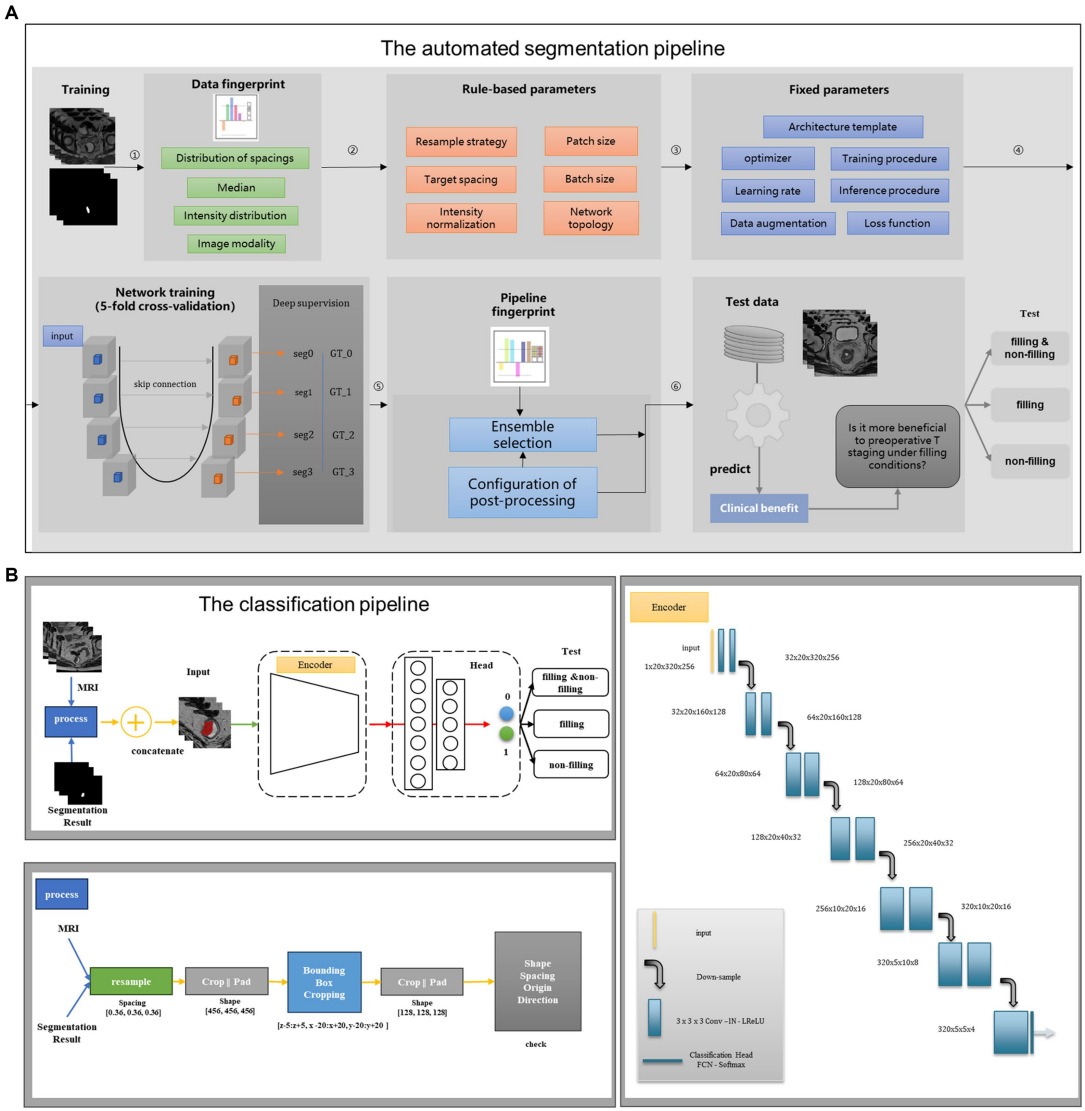
Structure diagram of the deep learning model. **(A)** The automated segmentation pipeline; **(B)** the classification pipeline. The first stage is to construct a segmentation flow chart to segment rectal cancer. The second stage is to build a classification flow chart and use the segmentation results of the first stage to do specific staging tasks.

Taking inspiration from these advancements, we incorporated a powerful and highly acclaimed network architecture, slightly modified by nn-UNet to be tailored for our rectal cancer data, as the backbone in Stage I of our study, where we trained it on a total of 265 cases. To adapt this network to our specific dataset and enable automatic segmentation of RC (Figure 2A), we rebuilt the training pipeline. We employed a larger dropout rate and more data augmentation strategy to prevent overfitting. In order to enhance the performance and generalizability of our model, we randomly divided the dataset with 5-fold cross-validation, implemented group normalization instead of batch normalization, and introduced larger convolution kernels. To evaluate the accuracy of the segmentation results, we calculated the dice similarity coefficient (DSC), the 95th percentile Hausdorff distance (HD95), and the average surface distance (ASD) between the automatically segmented images and GT images (26–28).

In contrast to the segmentation sub-task, the classification sub-task focuses on feature extraction after the convolution stage without the need to restore the features to their original size, which is one of the differences between the classification and segmentation tasks. Thus, in Stage II of our study, we designed an appropriate encoder as the backbone, which is the encoder of the 3D UNet, to extract features after convolution. For the output layer, we incorporated a multi-layer perception network to classify the T stages of RC. The flowchart of the classification task is depicted in Figure 2B. To facilitate T-stage classification in Stage II, we introduced a simple and easily manageable padding-cropping strategy. This involved utilizing the segmentation results obtained from Stage I and treating them as the input for the classification task, following the process outlined in Figure 2B. To construct our DL models, we divided the dataset into three categories based on the rectal filling status: model 1 trained on the complete training set of 265 cases, model 2 trained exclusively on rectal-filling cases, and model 3 trained using the non-filling dataset. The construction details of these models are provided in Supplementary material.

### Statistical analysis

To perform the statistical analysis, we employed two software tools: MedCalc (version 19.8, MedCalc Software, Mariakerke, Belgium) and the R package (version 4.1.3, Vienna, Austria). Normality testing of all continuous variables was conducted using the Kolmogorov–Smirnov test to assess their distribution. Categorical data were compared using either the Pearson Chi-square test or the Fisher’s exact test, depending on the expected cell counts. For continuous variables, presented as mean ± standard deviation, comparisons were made using either the Student’s *t*-test for normally distributed data or the Kruskal–Wallis H test for variables with non-normal distributions. To comprehensively evaluate the diagnostic performance of the T-staging classification models, we employed rigorous statistical techniques. Receiver operating characteristic (ROC) curve analysis and the confusion matrix were utilized to assess the models’ discriminative abilities in the independent test datasets. Essential performance measures, such as sensitivity, specificity, accuracy, positive predictive value (PPV), negative predictive value (NPV), positive likelihood ratio (PLR), and negative likelihood ratio (NLR), were determined to provide a comprehensive understanding of the models’ diagnostic values. Furthermore, to compare the classification models with subjective evaluation, we conducted net reclassification index (NRI) analysis. To gauge the clinical significance of the models, decision curve analysis (DCA) was performed, allowing us to calculate the net benefit. Statistical significance was established at a two-sided *p*-value less than 0.05, indicating strong evidence for significance.

## Results

### Patients’ characteristics

A comprehensive overview of patient demographic characteristics can be found in Table 1. After thorough evaluation, a total of 317 patients were included in the final analysis. Among them, 158 out of 317 cases (49.8%) underwent rectal filling, while the remaining 159 cases (50.2%) were in the non-filling group. Notably, there were no significant differences observed between these two cohorts of patient demographic characteristics (*p* > 0.05). The subsequent examination of the 52 test cases revealed an equal distribution of 26 cases each in both the filling and non-filling groups. Importantly, no statistically significant differences in T stage were detected between the filling and non-filling groups (T1-2/T3-4: 14/12 vs. 10/16, *p* = 0.404). Moreover, it is noteworthy that none of the cases exhibited positive circumferential resection margin (CRM) involvement.

**Table 1:** Pathological characteristics of patients.

Variables		Total	Rectal filling	Non-rectal filling	*p* value
		*N* = 317	*N* = 158	*N* = 159	
Gender					0.326
	Male	206 (65.0%)	98 (62.0%)	108 (67.9%)	
	Female	111 (35.0%)	60 (38.0%)	51 (32.1%)	
Age (years)		59.4 ± 12.0	58.3 ± 9.8	59.8 ± 10.3	0.185
BMI (kg/m^2^)		23.9 ± 3.4	23.8 ± 3.0	24.2 ± 3.3	0.260
Tumor location					0.161
	Upper	45 (14.2%)	20 (12.7%)	25 (15.7%)	
	Middle	190 (59.9%)	103 (65.2%)	87 (54.7%)	
	Lower	82 (25.9%)	35 (22.1%)	47 (29.6%)	
Differentiation					0.977
	High-Moderate	250 (78.9%)	124 (78.5%)	126 (79.2%)	
	Poor	67 (21.1%)	34 (21.5%)	33 (20.8%)	
T stage					0.078
	T1-2	180 (56.8%)	98 (62.0%)	82 (51.6%)	
	T3-4	137 (43.2%)	60 (38.0%)	77 (48.4%)	
N stage					0.971
	N0	222 (70.0%)	110 (69.6%)	112 (70.4%)	
	N1-2	95 (30.0%)	48 (30.4%)	47 (29.6%)	
Tumor deposit					0.816
	Negative	242 (76.3%)	122 (77.2%)	120 (75.5%)	
	Positive	75 (23.7%)	36 (22.8%)	39 (24.5%)	
Lymphovascular invasion					0.184
	Negative	190 (59.9%)	101 (63.9%)	89 (56.0%)	
	Positive	127 (40.1%)	57 (36.1%)	70 (44.0%)	
Perineural invasion					0.197
	Negative	152 (47.9%)	82 (51.9%)	70 (44.0%)	
	Positive	165 (52.1%)	76 (48.1%)	89 (56.0%)	
Tumor budding					0.669
	Negative	204 (64.4%)	104 (65.8%)	100 (56.6%)	
	Positive	113 (35.6%)	54 (34.2%)	59 (43.4%)	
CEA^*^					0.061
	Negative	177 (55.8%)	97 (61.4%)	80 (50.3%)	
	Positive	140 (44.2%)	61 (38.6%)	79 (49.7%)	
CA19-9^*^					0.593
	Negative	254 (80.1%)	129 (81.6%)	125 (78.6%)	
	Positive	63 (19.9%)	29 (18.4%)	34 (21.4%)	

*Preoperative blood samples. BMI, body mass index; CEA, carcinoembryonic antigen; CA19-9, carbohydrate antigen 19-9.

### Automatic segmentation results

**Figure 3:**
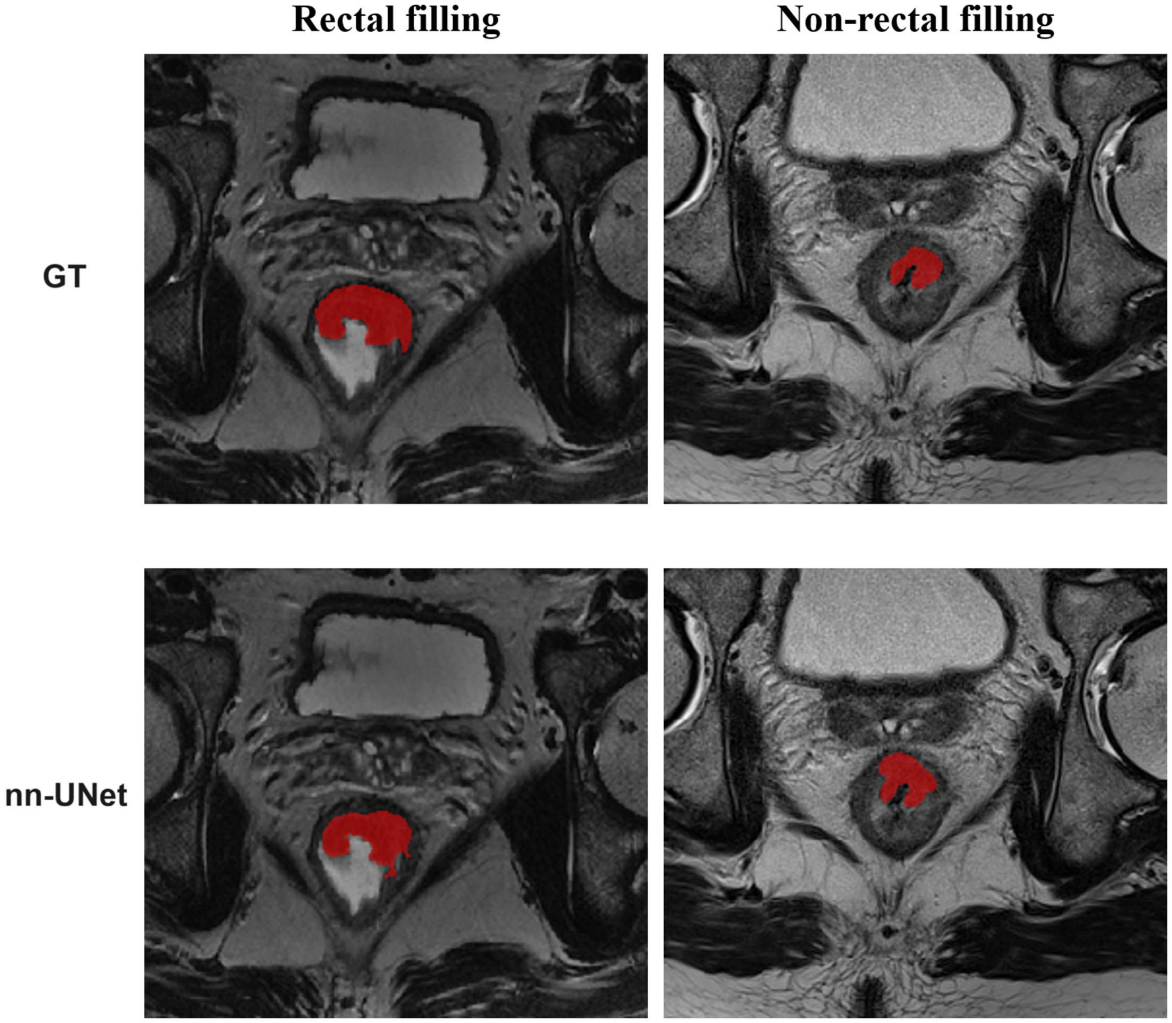
Representative diagram of automatic segmentation.

In Stage I, we developed a segmentation pipeline utilizing DL models, which can be succinctly referred to as nn-UNet. These automatic segmentation models exhibited exceptional performance in the test datasets, as illustrated in Figure 3. For the overall test dataset, the median values of DSC, HD95, and ASD were 0.835, 2.236 mm, and 0.647 mm, respectively. In the rectal-filling cases, the median values were 0.862 for DSC, 2.118 mm for HD95, and 0.584 mm for ASD. Conversely, in the non-filling cases, the median values were 0.807 for DSC, 3.000 mm for HD95, and 0.879 mm for ASD. Notably, the DSC and ASD values were higher in the rectal-filling dataset (*p* = 0.006 and *p* = 0.017, respectively). The detailed results of the automatic segmentation are presented in Table 2 and Supplementary Figure S1.

**Table 2:** Automatic segmentation results.

Test data sets	Total	Rectal filling	Non-rectal filling	*p* value^*^
DSC	0.835 (0.779, 0.871)	0.862 (0.817, 0.879)	0.807 (0.719, 0.850)	0.006
HD95 (mm)	2.236 (2.000, 3.742)	2.118 (1.732, 2.450)	3.000 (2.000, 5.385)	0.127
ASD (mm)	0.647 (0.516, 1.034)	0.584 (0.440, 0.737)	0.879 (0.616, 1.332)	0.017

Median (IQR).

^*^Kruskal–Wallis rank sum test.

DSC, dice similarity coefficient; HD95, the 95th percentile Hausdorff distance; ASD, average surface distance.

### Classification performance

**Figure 4:**
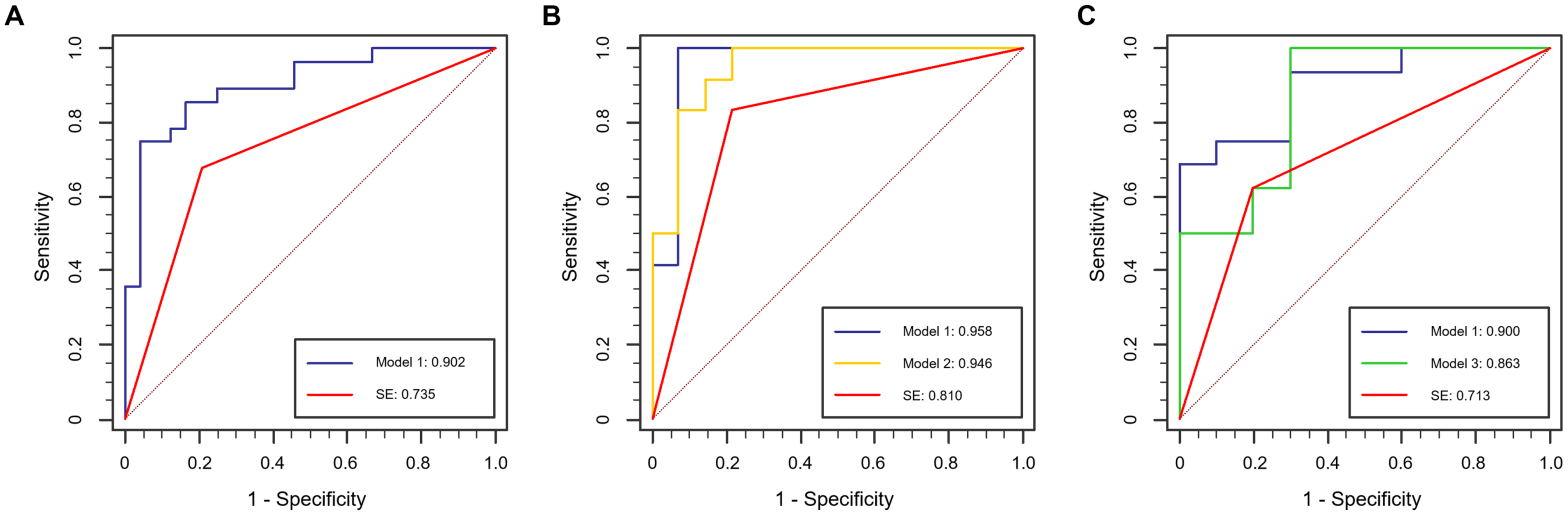
ROC curves in the test dataset. **(A)** Total test dataset; **(B)** rectal-filling test dataset; **(C)** non-filling test dataset.

In Stage II, concentrating on T-staging classification, the subjective evaluation yielded area under the curve (AUC) values of 0.735, 0.810, and 0.713 for the total, filling, and non-filling test datasets, respectively. The corresponding accuracies were 0.731, 0.808, and 0.692. Interobserver agreement for MR T-staging among the three radiologists is presented in Supplementary Table S2. Notably, the DL models outperformed the subjective evaluation across all test datasets. Model 1 achieved AUC values of 0.902, 0.958, and 0.900 for the total, filling, and non-filling test datasets, respectively. The accuracies were 0.846, 0.962, and 0.808, respectively. Model 2, trained on rectal-filling cases, exhibited an AUC of 0.946 and an accuracy of 0.885 in the filling test dataset. Model 3, trained exclusively on non-filling cases, demonstrated an AUC of 0.863 and an accuracy of 0.885 in the non-filling test dataset. Comprehensive ROC analyses are presented in Table 3, and the corresponding curves are depicted in Figure 4.

**Table 3:** ROC curve analysis and comparison in the test dataset.

	Total		Filling			Non-filling		
	Model 1	SE	Model 1	Model 2	SE	Model 1	Model 3	SE
AUC	0.902	0.735	0.958	0.946	0.810	0.900	0.863	0.713
95% CI	0.818–0.985	0.594–0.848	0.874–0.999	0.865–0.999	0.653–0.966	0.784–0.999	0.706–0.999	0.533–0.892
Sensitivity	0.750	0.679	1.000	1.000	0.833	0.688	1.000	0.625
Specificity	0.958	0.792	0.929	0.786	0.786	1.000	0.700	0.800
Accuracy	0.846	0.731	0.962	0.885	0.808	0.808	0.885	0.692
PLR	18.000	3.257	14.000	4.667	3.889	Inf.	3.333	3.125
NLR	0.261	0.406	0.000	0.000	0.212	0.313	0.000	0.469
PPV	0.955	0.792	0.923	0.800	0.769	1.000	0.842	0.833
NPV	0.767	0.679	1.000	1.000	0.846	0.667	1.000	0.571
NRI^*^	0.238		0.310	0.167		0.263	0.275	

SE, subjective evaluation; NPV, negative predictive value; PPV, positive predictive value; NLR, negative likelihood ratio; PLR, positive likelihood ratio.

^*^NRI, net reclassification index, compared to SE.

Model 1: trained on total training datasets; Model 2: trained on rectal-filling datasets; Model 3: trained on non-filling datasets.

### Model comparison and clinical utility

Compared to subjective evaluation for RC T-staging, NRIs of DL-models were 0.167 to 0.310, demonstrating an improved clinical utility in all datasets (Table 3).

**Figure 5:**
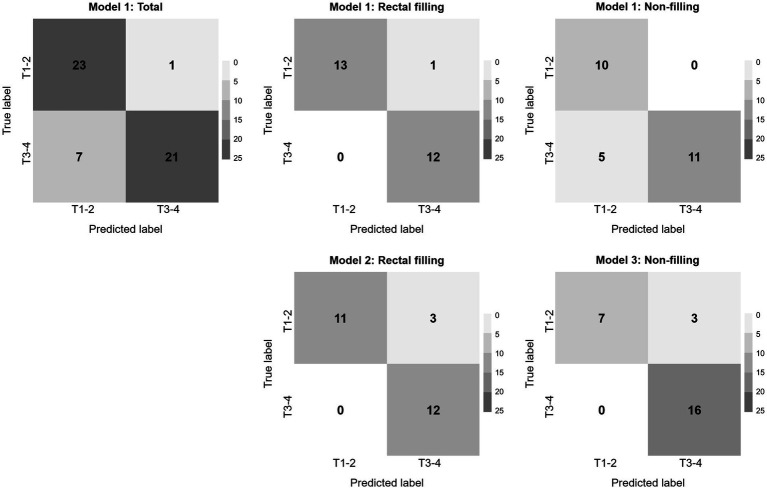
Confusion matrices.

**Figure 6:**
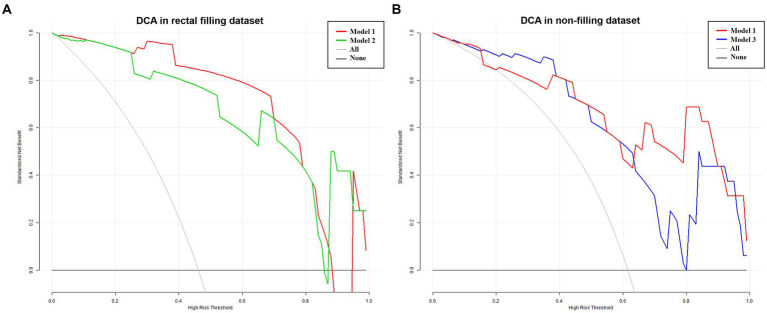
DCA in filling and non-filling test datasets. Results for the rectal-filling dataset. **(A)** The net benefit analysis showed that for *p* probability thresholds ranging from 0.25 to 0.88 in the test dataset, Model 1 provided greater benefits compared to Model 2 assessment. Moreover, Model 1 exhibited larger net benefits when compared to all/no intervention methods. **(B)** Results for the non-filling dataset. The net benefit analysis demonstrated that for P probability thresholds ranging from 0.42 to 0.91 in the test dataset, Model 1 yielded additional benefits compared to Model 3 assessment. Furthermore, Model 1 showed larger net benefits when compared to all/no intervention methods.

Considering the influence of rectal filling or non-filling, the confusion matrix highlighted the superior classification performance of Model 1 in the rectal-filling dataset compared to Model 1 in the non-filling dataset. Likewise, the performance of Model 2 in the filling dataset outperformed that of Model 3 in the non-filling dataset (Figure 5). The net clinical advantage of Model 1 over Model 2 in the rectal-filling dataset and Model 1 over Model 3 in the non-filling dataset is illustrated by the DCA chart (Figure 6). Overall, Model 1 in the rectal-filling dataset demonstrated notably improved diagnostic performance.

## Discussion

In this study, we developed an advanced and automated segmentation model based on nn-UNet to achieve precise segmentation of rectal adenocarcinomas from T2W images, particularly in the rectal-filling dataset. Subsequently, we constructed DL-based classification models that exhibited significantly improved performance in T-staging classification compared to subjective evaluation for RC cases. Notably, Model 1, trained on the total training dataset, demonstrated higher AUC and accuracy in the rectal-filling cohort. To the best of our knowledge, this is the first investigation into the impact of rectal filling on DL models, highlighting its influence on classification performance.

In the current landscape of medical practice, rectal MRI has been widely endorsed as the optimal approach for preoperative T-staging in RC. However, its diagnostic accuracy is compromised by the connective tissue hyperplastic response in the surrounding rectal mesenteric fat, leading to indistinct tumor boundaries. This limitation of traditional MRI techniques in distinguishing between T2 and T3 stages has been well-documented in previous studies (3–5), and our own results align with these observations. Our study conducted a comprehensive ROC analysis, revealing that the subjective discrimination of preoperative T-stage by radiologists was significantly inferior to the proposed DL model. The accuracies of radiologists’ assessments ranged from 69.2 to 80.8%. Additionally, the net reclassification index (NRI) analysis demonstrated improved classification performance achieved by employing DL approaches, while decision curve analysis (DCA) highlighted the favorable clinical usefulness of these models. These findings can be attributed to the inherent challenges faced by radiologists in accurately interpreting irregular tumor shapes and blurred boundaries. Therefore, the accurate identification and precise segmentation of lesions serve as crucial prerequisites for future research endeavors aimed at advancing preoperative evaluation and staging methodologies in RC.

The routine utilization of manual or semi-manual segmentation methods is often plagued by inherent challenges, including their arduous and time-consuming nature, as well as their heavy reliance on operator expertise (16, 17). In recent years, several studies have explored the application of 2D convolutional neural network (CNN) models for the discrimination of T2 and T3 stages using 2D MR images (29, 30). However, these approaches introduce an additional burden on radiologists, as they require manual selection of a representative slice (2D) from each MR volume (3D). This manual selection step adds complexity and potential subjectivity to the process. Hou et al. conducted a study where they developed a DL model using 3D T2W images, achieving an impressive AUC value of 0.869 (31). However, it is important to note that the segmentation process in their research was carried out manually, which may introduce subjectivity and potential variability. In a separate study by Wei et al., a multi-parametric MR image fusion model was employed, achieving an AUC of 0.854. This approach involved the manual determination of the location and size of a 3D bounding box containing the tumor (32).

In Stage I, we successfully developed an automatic segmentation model for rectal adenocarcinomas using a 3D nn-UNet architecture. As a standardized and dataset-agnostic framework, nnU-Net was proposed as a robust and powerful tool for medical image segmentation. The results demonstrated impressive performance, with median values of 0.807–0.862 for DSC, 2.118–3.000 for HD95, and 0.584–0.879 for ASD in the test dataset. To enhance the robustness and generalization of the model while avoiding overfitting, we employed a data augmentation strategy along with 5-fold cross-validation. Furthermore, two experienced radiologists carefully examined the visualizations of the segmentation results, and no noticeable segmentation errors were detected. The implementation of this method bears the potential to serve as a viable replacement for the prevailing manual segmentation method, which is notorious for its time-consuming nature and lack of reproducibility. Subsequently, we conducted additional evaluations on the total test dataset, as well as the rectal-filling and non-filling datasets within the test set. Our findings revealed that the DSC and ASD values were significantly better in the rectal-filling datasets compared to both the total datasets and the non-filling datasets (*p* = 0.006 and *p* = 0.017, respectively). These results suggest that the model exhibited a tendency towards better performance and metrics in rectal-filling cases.

In the Stage II of this study, we introduced a 3D CNN to classify RC lesions as T1-2 or T3-4 stages on HR-T2WI. For the classification models, we utilized widely-used UNet-like Encoder–Decoder architectures. We recognized that directly inputting the original images into the models would make it challenging to distinguish between T1-2 and T3-4 stages, as the models might concentrate on areas other than the cancer of interest. To address this concern, we devised a novel approach using the information from the automated segmentation results obtained in Stage I. We combined the original MRI of RC with its corresponding segmentation result, incorporating them as the complete input. This approach involved employing a region-of-interest cropping strategy, as mentioned earlier. Our initial experiments demonstrated the effectiveness and correctness of this approach compared to solely using the original MRI of RC with a center-cropping strategy. We believe that the center-cropping strategy may not accurately select the cancerous region, as the cancer might not always be located in the center of every image. Although expanding the cropping area could be considered, it would introduce redundant information that is not helpful. Therefore, our cropping strategy, as described above, represents a promising approach for precisely selecting the cancerous region in each original image.

A distinctive feature of our study was its groundbreaking exploration and validation of DL models for preoperative T staging in RC, with a particular focus on the influence of rectal filling. To the best of our knowledge, this was the first endeavor to address this specific aspect, shedding new light on the application of DL in this context.

Our study encompassed the evaluation of automatic segmentation models for rectal adenocarcinomas across three distinct datasets: the total dataset, the rectal-filling dataset, and the non-filling dataset. Following this, three DL models were trained using these datasets to explore their performance. Through a comprehensive analysis involving segmentation results, ROC evaluation, confusion metrics, and DCA, a noteworthy finding emerged: Model 1 exhibited superior performance specifically in the rectal-filling dataset. These results underscore the additional benefits conferred by the use of rectal contrast material in DL models. Previous research has already demonstrated the advantages of rectal filling, including improved lesion visualization and enhanced evaluation of tumor penetration on MRI (2). Furthermore, our previous study has corroborated the value of rectal-filling in accurately delineating rectal lesions and distinguishing them from normal rectal tissue, thus facilitating precise segmentation (6). This likely explains the higher performance observed in the DL model trained on the rectal-filling dataset compared to the non-filling dataset.

Despite the notable contributions of our study, several limitations warrant consideration. Firstly, our dataset consisted solely of HR-T2WI of RC, lacking the inclusion of other imaging modalities. Moreover, being a retrospective single-center study, potential selection biases may have influenced our findings. Thus, for further validation and to enhance the generalizability of our results, larger datasets and multi-center studies incorporating diverse imaging modalities are necessary. Secondly, it is crucial to acknowledge that factors, such as extramural vascular invasion (EMVI), lymph node metastasis (LNM), and mesorectal fascia (MRF) significantly influence the prognosis and survival of RC patients (2–4, 33). While the impact of rectal luminal distention on DL-models pertaining to MRF, EMVI, and LNM remains a topic of debate, further investigation is essential to elucidate these associations comprehensively. Thirdly, an important consideration is the generalizability of our findings to lesions that have undergone neoadjuvant treatment, as this aspect remains elusive and requires further clarification. Finally, the current investigation primarily employed CNN-based models, which are known to perform well with small datasets. However, we did not explore the use of transformer-based models, which are better suited for larger datasets. Therefore, future research should encompass the incorporation of transformer-based models to leverage their potential in handling larger datasets effectively.

## Conclusion

Leveraging high-resolution rectal MR imaging, we developed a DL-based segmentation model to automatically extract the region of RC. Subsequently, we constructed DL-based classification models to explore an innovative approach for preoperative T-staging of RC using DL networks. Through a comprehensive comparison, we observed that the DL models exhibited superior predictive capabilities compared to subjective evaluation, particularly in distinguishing between T1-2 and T3-4 stages in the test dataset with rectal-filling. These findings strongly indicate that the DL model, augmented by rectal-filling, holds significant potential as an optimal tool for guiding clinical practice in the preoperative T-staging of RC patients.

## Data availability statement

The raw data supporting the conclusions of this article will be made available by the authors, without undue reservation.

## Ethics statement

The studies involving humans were approved by the Shanghai Changhai Hospital, Naval Medical University. The studies were conducted in accordance with the local legislation and institutional requirements. Written informed consent for participation was not required from the participants or the participants’ legal guardians/next of kin in accordance with the national legislation and institutional requirements.

## Author contributions

CT: Data curation, Formal analysis, Writing – original draft. XM: Data curation, Methodology, Software, Writing – review & editing. HL: Data curation, Writing – review & editing. QW: Data curation, Writing – review & editing. CS: Conceptualization, Writing – review & editing. YY: Conceptualization, Formal analysis, Investigation, Methodology, Software, Supervision, Writing – review & editing. FS: Conceptualization, Formal analysis, Funding acquisition, Investigation, Methodology, Project administration, Software, Supervision, Writing – review & editing.

## Footnotes

^1^ http://www.itksnap.org/
