# Supplementary material for: Deep learning models for preoperative T-stage assessment in rectal cancer using MRI: exploring the impact of rectal filling
Source: Front Med (Lausanne). 2023 Nov 29;10:1326324. doi: 10.3389/fmed.2023.1326324 (PMC10722089; doi:10.3389/fmed.2023.1326324)
Supplement: Supplementary file 1 [file Data_Sheet_1.docx]

**Supplementary files**

**Details of deep learning model**

**Data augmentation**

Data Augmentation [1], as one of the essential methods of enlarging the diversity of the dataset, is the key to guiding and teaching the network to be general and robust, when only a few training samples are available, especially in the medical field. This strategy mainly involves random transformation of the original data with a certain probability when the amount of data is small to prevent the model from overfitting. In the case of MRI medical images, we primarily need rotation, scaling, gaussian noise, gaussian blur, brightness, contrast adjustment, simulation of low resolution, gamma augmentation, mirroring all the like to be applied during the training process. The main purpose is to enhance the sample data in each batch of the training process. This means that the data augmentation for the data in the batch in each iteration and each epoch is different. Of course, there is a high probability that the original data will be maintained without data enhancement.

**Software, hardware, and hyperparameters**

We implemented all the experiments based on Python 3.8, Pytorch 1.12.0, and Ubuntu 20.04. All training procedures have been run on a single NVIDIA TITAN RTX with 24GB memory. For Stage II, the segmentation task, the initial learning rate was set to 0.01 with a “poly” decay strategy as described in Equation 1. The network parameters are optimized with Stochastic Gradient Descent (SGD) [2] with a momentum of 0.99. The weight decay is set to 3e-5. The number of training epochs (i.e., max_epochs) is 1000 and contains 250 iterations every epoch.

$lr=initial_{lr}\times{(1-\frac{epoch}{max\_epoch})}^{0.9}$. (1)

For Stage II, the classification task, the learning rate was set to 0.0001, and the network parameters are optimized with Adam [3], which was scheduled by StepLR. The number of training epochs is 300. Last but not least, the fixed seed was set to be 42 for all the experiments.

**Supplementary Objective Function**

One of the most vital components of deep learning models is the objective function, also known as the loss function, which could measure the gap between the output of the deep learning models and the offered ground truth.

In Stage I, we used a joint cross-entropy [4] and soft dice loss to be the supervised loss function, which is given as in Equation 2:

$\mathcal{L}_{seg}\{h, w,d\}=\frac{1}{2}[\mathcal{L}_{CE}\left\{ h,w,d \right\}+\mathcal{L}_{Dice}\left\{ h,w,d \right\}]$. (2)

Cross Entropy Loss, which can be traced back to the information entropy proposed by Shannon, is a common basic classification loss function in machine learning. It is used to determine how close the actual output is to the desired output.

Dice Loss, from the original V-Net paper [5], makes the best use of the Dice Similarity Coefficient (DSC) which is the most frequently used metric in medical image competitions. DSC is a set similarity metric, used to calculate the similarity between two samples and often used in medical segmentation. The value threshold is between 0 and 1. The best result of segmentation is 1, and the worst result is 0. Later, Dice Loss, described as $Dice Loss = 1 - DSC$, plays an extraordinary role till now.

To precisely extract cancer in Stage I, we also added deep supervision during the training stage. More specifically, the output of each stage in the decoder would be passed to the final expanding block, where cross-entropy loss and dice loss would be applied. Practically, we down-sampled the ground truth segmentation mask so as to match the resolution of the model’s prediction. Therefore, the final training objective function is the sum of all losses at four resolutions in Equation 3.

$\mathcal{L}_{all}=\delta_{1}\mathcal{L}_{seg}\left\{ h,w,d \right\}+\delta_{2}\mathcal{L}_{seg}\left\{ \frac{h}{4},\frac{w}{4},\frac{d}{2} \right\}+\delta_{3}\mathcal{L}_{seg}\left\{ \frac{h}{8},\frac{w}{8},\frac{d}{4} \right\}+\delta_{4}\mathcal{L}_{seg}\left\{ \frac{h}{16},\frac{w}{16},\frac{d}{8} \right\}$ (3)

Here, $\delta_{\{1, 2,3\}}$ denotes the magnitude factor for losses in different resolution. Practically, $\delta_{\{1, 2,3\}}$ halves with each decrease in resolution, which means $\delta_{2}=\frac{\delta_{1}}{2}$, $\delta_{3}=\frac{\delta_{1}}{4}$, and $\delta_{4}=\frac{\delta_{1}}{8}$. At last, all the weight factors are normalized to 1.

In Stage II, we used a joint loss consisting of focal loss, and L_1_ smooth loss, to be the objective function, which is given in Equations 4, 5, 6:

$\mathcal{L}_{focal}\left( p \right)= -\left( 1-p \right)^{\gamma}log(p)$ (4)

$\mathcal{L}_{cls}\{w,h,d\}=\alpha\mathcal{L}_{focal}\{w,h,d\}+\beta\mathcal{L}_{l1s}\{w,h, d\}$ (5)

$\alpha+\beta=1$ (6)

The focal loss [6], first introduced by Kaiming He, aiming to solve the extreme sample imbalance during training, is a generalization of cross-entropy loss with an additional down-weighting parameter for when the prediction is close to the ground truth. Where $p$ is the soft-max probability.

The $L_{1}$ smooth loss [7], introduced by Ross Girshick, is a robust $L_{1}$ loss that is less sensitive to outliers than the $L_{2}$ loss. That is to say, compared to L_2_ Loss [8], it is more robust to outliers, the gradient change is relatively small, and it is not easy to run away during training.

The hyper-parameters $\alpha$ and $\beta$ represent the different weights of the loss. Empirically, we choose $\alpha=0.5, \beta=0.5.$

**Metrics on evaluation**

We use DSC, HD95 and ASD to evaluate the accuracy of segmentation in the experiments. For a given semantic class, we assume $G_{i}$ and $P_{i}$ denote the ground truth and prediction values for voxel $i$ and $G^{'}$and $P^{'}$ denote ground truth and prediction surface point set, respectively. The DSC, HD95 and ASD metrics are defined as:

DSC ($G, P$) = $\frac{2\sum_{i=1}^{I} G_{i}P_{i}}{\sum_{i=1}^{I} G_{i}+\sum_{i=1}^{I} P_{i}}$, (7)

HD95 ($G^{'},P^{'}$) = $max\underset{g^{'}\in G^{'}}{\{max} \min_{p^{'}\in P^{'}} \left\| g^{'}-p^{'} \right\|,\max_{p^{'}\in P^{'}} \min_{g^{'}\in G^{'}} \left\| p^{'}-g^{'} \right\|\}$, (8)

The 95% HD uses the 95th percentile of the distances between ground truth and prediction surface point sets. As a result, the impact of a very small subset of outliers is minimized when calculating HD.

ASD is a measurement of the average of all Euclidean distances between ground truth and the prediction. It could be defined as below:

ASD = ($G^{''},P^{''}$) = $\sum_{g^{''}\in G^{''}} \frac{\min_{p^{''}\in P^{''}} d(g^{''}, p^{''})}{\left\| G^{''} \right\|}$, (9)

where $d(g^{''}, p^{''})$ is a 3D matrix consisting of the Euclidean distances between the ground truth and the prediction.

The best result of DSC is 1, and the worst result is 0. The higher the score is, the larger the union of the prediction and the ground truth is. The unit of HD95 and ASD is millimeter. The smaller the distance is, the closer the prediction is to the ground truth.

**Supplemental Table S1. Oblique axial high-resolution T2WI sequence parameters**

|  | TR/TE (m/s) | Matrix | FOV  (mm) | Slice thickness/gap (mm) | Bandwidth (Hz) /flip angle (°) | Acquisition times |
| --- | --- | --- | --- | --- | --- | --- |
| Siemens Skyra  3.0 T | 4000/108 | 320×320 | 180×180 | 3/0 | 108/160 | 4 min 10 sec |
| GE Discovery 750w 3.0 T | 7845/109 | 352×352 | 200×200 | 4/0.5 | 62.5/110 | 2 min 37 sec |
| GE Signa  HDX 3.0 T | 4000/113 | 288×288 | 180×180 | 3/0 | 83.3/90 | 5 min 04 sec |

**Supplemental Table S2. Interobserver agreement for** **MR T-staging among the three radiologists**

| **Radiologist 1 - 2**  ***Kappa* value (95% CI)** | **Radiologist 1 - 3**  ***Kappa* value (95% CI)** | **Radiologist 2 - 3**  ***Kappa* value (95% CI)** | **All Radiologists**  **ICC (95% CI)** |
| --- | --- | --- | --- |
| 0.751 (0.638 - 0.865) | 0.863 (0.778 - 0.948) | 0.880 (0.801 - 0.959) | 0.831 (0.780 - 0.874) |

ICC, intraclass correlation coefficient

**Supplementary Figure S1. Comparison between automatic segmentation results**

**
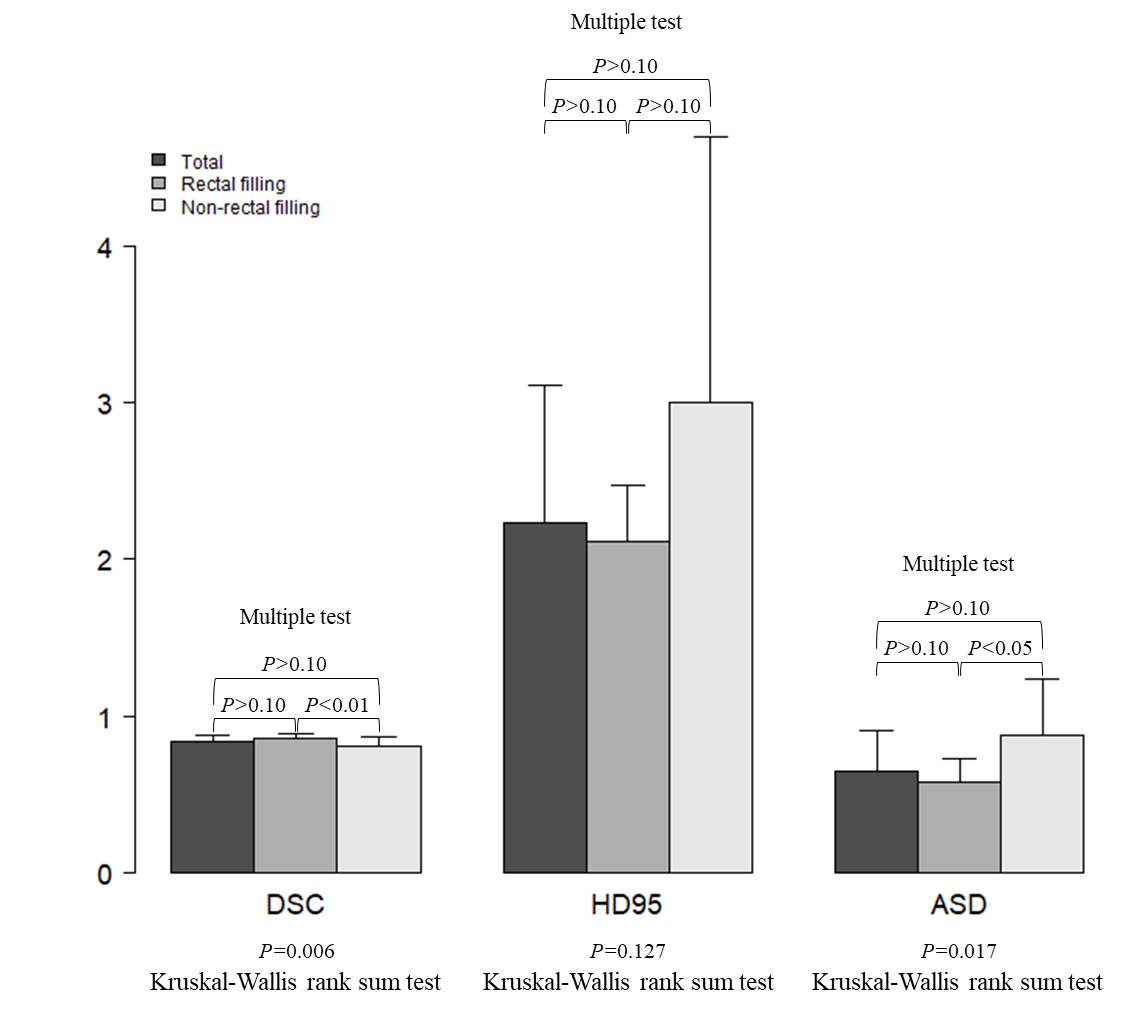
**

**Supplemental reference**

1. Image Data Augmentation for Deep Learning: A Survey

2. Song, Shuang, Kamalika Chaudhuri, and Anand D. Sarwate. "Stochastic gradient descent with differentially private updates." *2013 IEEE global conference on signal and information processing*. IEEE, 2013.

3. Kingma, Diederik P., and Jimmy Ba. "Adam: A method for stochastic optimization." *arXiv preprint arXiv:1412.6980* (2014).

4. Zhang, Zhilu, and Mert Sabuncu. "Generalized cross entropy loss for training deep neural networks with noisy labels." *Advances in neural information processing systems* 31 (2018).

5. Milletari, Fausto, Nassir Navab, and Seyed-Ahmad Ahmadi. "V-net: Fully convolutional neural networks for volumetric medical image segmentation." *2016 fourth international conference on 3D vision (3DV)*. Ieee, 2016.

6. Lin, Tsung-Yi, et al. "Focal loss for dense object detection." *Proceedings of the IEEE international conference on computer vision*. 2017.

7. Girshick, Ross. "Fast r-cnn." *Proceedings of the IEEE international conference on computer vision*. 2015.

8. Barron, Jonathan T. "A general and adaptive robust loss function." *Proceedings of the IEEE/CVF Conference on Computer Vision and Pattern Recognition*. 2019.
